# On-site bundled rapid HIV/HCV testing in substance use disorder treatment programs: study protocol for a hybrid design randomized controlled trial

**DOI:** 10.1186/s13063-016-1225-4

**Published:** 2016-03-03

**Authors:** Jemima A. Frimpong, Thomas D’Aunno, David C. Perlman, Shiela M. Strauss, Alissa Mallow, Diana Hernandez, Bruce R. Schackman, Daniel J. Feaster, Lisa R. Metsch

**Affiliations:** Department of Health Policy and Management, Mailman School of Public Health, Columbia University, New York, USA; Robert F. Wagner Graduate School of Public Service, New York University, New York, USA; Mount Sinai Beth Israel; Ichan School of Medicine at Mount Sinai, New York, USA; College of Nursing, New York University, New York, USA; Montefiore Health System, New York, USA, New York, USA; Department of Sociomedical Sciences, Mailman School of Public Health, Columbia University, New York, USA; Department of Healthcare Policy and Research, Weill Cornell Medical College, New York, USA; Division of Biostatistics, Department of Public Health Sciences, Miller School of Medicine, University of Miami, Miami, USA

**Keywords:** HIV testing, HCV testing, Bundled HIV/HCV testing, Hybrid intervention-implementation research, Intervention adaptation, Substance use disorder treatment, Adoption of evidence-based practices, Implementation research

## Abstract

**Background:**

More than 1.2 million people in the United States are living with human immunodeficiency virus (HIV), and 3.2 million are living with hepatitis C virus (HCV). An estimated 25 % of persons living with HIV also have HCV. It is therefore of great public health importance to ensure the prompt diagnosis of both HIV and HCV in populations that have the highest prevalence of both infections, including individuals with substance use disorders (SUD).

**Methods/design:**

In this theory-driven, efficacy-effectiveness-implementation hybrid study, we will develop and test an on-site bundled rapid HIV/HCV testing intervention for SUD treatment programs. Its aim is to increase the receipt of HIV and HCV test results among SUD treatment patients. Using a rigorous process involving patients, providers, and program managers, we will incorporate rapid HCV testing into evidence-based HIV testing and linkage to care interventions. We will then test, in a randomized controlled trial, the extent to which this bundled rapid HIV/HCV testing approach increases receipt of HIV and HCV test results. Lastly, we will conduct formative research to understand the barriers to, and facilitators of, the adoption, implementation, and sustainability of the bundled rapid testing strategy in SUD treatment programs.

**Discussion:**

Novel approaches that effectively integrate on-site rapid HIV and rapid HCV testing are needed to address both the HIV and HCV epidemics. If feasible and efficacious, bundled rapid HIV/HCV testing may offer a scalable, potentially cost-effective approach to testing high-risk populations, such as patients of SUD treatment programs. It may ultimately lead to improved linkage to care and progress through the HIV and HCV care and treatment cascades.

**Trial registration:**

ClinicalTrials.gov: NCT02355080. (30 January 2015)

## Background

More than 1.2 million people in the United States are living with HIV, with an estimated 50,000 persons newly infected each year [1]. Approximately 3.2 million people are chronically infected with HCV [2, 3]. HIV/HCV co-infection is common: up to 25 % of the people infected with HIV in the US also have HCV [4, 5]. The prevalence of HIV and HCV in the US is particularly high among persons with substance use disorders (SUDs). Approximately, 25 % of HIV/AIDS cases are directly or indirectly related to injection drug use, [6] and the prevalence of HCV among people who inject drugs (PWID) ranges from 35 to 65 % [2, 7, 8]. HIV and HCV are also common among persons who use but do not inject drugs (for example, intranasal use of cocaine or heroin) [9]. Among those infected with HIV and/or HCV, significant numbers of people are not aware of their infection and are 1) more likely to transmit infection(s); 2) less likely to benefit from early treatment; and 3) more prone to morbidity, mortality, and complications due to co-infection [10–12]. The Centers for Disease Control and Prevention (CDC) recommend routine HIV and HCV testing for people with a history of drug use [13–15].

The availability of both HIV and HCV testing in SUD treatment programs is limited. Fewer than half of U.S. SUD treatment programs offer HIV testing on-site to their patients [10, 16–20]. The availability of on-site HCV testing even declined from 53 % in 2005 to 34 % in 2011 in opioid treatment programs [21]. The limited availability of on-site testing is related to gaps in the capacity of SUD treatment programs to conduct laboratory-based HIV or HCV testing. [18, 22] In recent years, SUD treatment programs have increasingly offered off-site referrals for HIV and/or HCV testing to their patients, instead of incurring large fixed costs required to make laboratory-based HIV testing available on-site [23, 24]. In addition, laboratory-based HIV or HCV testing methods do not produce immediate results, and thus require patients to return to the clinic for their test results. Unfortunately, few patients follow up on referrals for off-site testing [24, 25] or return for their test results [26–29].

Rapid testing assays, introduced more than 10 years ago for HIV and in 2010 for HCV, have the potential to address several of these barriers to the scale up of HIV/HCV testing in SUD treatment programs [30–32]. Rapid tests for HIV and HCV use blood from a finger stick or oral fluid from a swab and can be conducted under a Clinical Laboratory Improvement Agreement (CLIA) Certificate of Waiver. [33] They do not require extensive laboratory facilities and can be performed without a doctor, nurse, or phlebotomist. They are also highly accurate [26, 31, 34] and yield preliminary results in no more than 20 minutes, which allows testing and result notification to occur during the same visit.

Despite several calls for the integration of HIV and HCV services [35–41], SUD treatment programs have not yet adopted a rapid HIV/HCV testing “bundle” for several reasons [26]. First, offering both tests might be time consuming for staff that already faces a significant workload. Offering on-site bundled HIV/HCV testing thus requires streamlined testing procedures to facilitate integration and reduce constraints on service delivery. Second, some patients may become particularly distressed if they learn that they are infected with both HIV and HCV in the same visit. Post-test counseling procedures adapted to the provision of bundled testing must therefore be developed. Third, in the past, HCV treatments were arduous, ineffective, and associated with serious side effects. The recent introduction of new, safe, and highly efficacious treatment options for HCV address these issues [42] and suggest that HCV testing may now present significantly more clinical benefits to patients. Fourth, treatments for HCV remain expensive. However, increased access to health insurance through the Affordable Care Act (ACA) [43] may present opportunities for increasing access to HCV treatment and care for persons with SUDs who may have previously lacked health insurance [44, 45].

### Study objective

The overarching objective of this study is to develop and test an on-site bundled rapid HIV and HCV testing strategy to facilitate the timely diagnosis and receipt of test results for both HIV and HCV among persons with SUDs.

## Methods/design

### Study design

This efficacy-effectiveness-implementation hybrid study consists of three distinct phases. Hybrid study designs combine features of clinical trials, which examine the efficacy, effectiveness, and the implementation research, which examines the adoption and sustainability of service delivery processes [46, 47]. Hybrid designs are postulated to advance the adoption and use of evidence-based practices and enhance the potential benefits of effective interventions [48–50]. In the “developmental phase” of this study, we will produce a protocol for the provision of on-site bundled rapid HIV/HCV testing in SUD treatment programs. This will be followed by a “trial” phase, during which we will test the efficacy of the on-site bundled rapid HIV/HCV testing approach in increasing receipt of HIV and HCV test results among SUD patients. Finally, during a “translation” phase, we will conduct formative research on the barriers and facilitators of the diffusion, adoption, implementation, and sustainability of on-site bundled rapid HIV/HCV testing in SUD treatment programs. Overall, we will develop and evaluate the efficacy of the bundled rapid testing intervention and explore the preliminary effectiveness and implementation considerations related to intervention scale up. This study protocol follows the SPIRIT statement (http://www.spirit-statement.org/spirit-statement/).

#### Conceptual/theoretical framework

To guide our approach to “bundling” the offer of on-site rapid HIV and rapid HCV testing, we use an enhanced version of ADAPT-ITT, [51–53] a framework used to transfer evidence-based HIV interventions (HIV EBIs) to new relevant populations. ADAPT-ITT builds on a sequence of Assessment-Decision-Administration-Production-Topical experts (ADAPT) -Integration-Training-Testing (ITT) and culminates in a rigorous randomized controlled trial of the impact of the newly developed intervention on short-term outcomes (for example, receipt of HIV and HCV test results). Because we use ADAPT-ITT to add a multi-infection component to existing HIV EBIs, rather than to transfer EBIs across relevant populations, we modify some of the framework’s procedures. The “decision” phase of ADAPT-ITT is replaced by a “design” phase, during which we will address key questions related to the structure and content of the testing session. We also enhance the training component of the model by integrating a standardized patients (SP) approach, an objective structured clinical education procedure for training, evaluating and providing feedback to providers to ensure the effective completion of clinical tasks [54, 55].

To enable a broad scale up of on-site bundled HIV/HCV testing, we add a final “translational” step to the ADAPT-ITT process. We refer to this extended framework as “ADAPT-IT^3^.” Our translational approach is guided by a rich, interdisciplinary understanding of the “Diffusion of Innovation” (DOI) theory outlined by Rogers [56, 57]. DOI is defined as the process through which an innovation is spread over time via communication (information, attitudes, etcetera) among members of a social system (patients and organizations) [57]. The goal of DOI is to advance an innovation from adoption, through implementation, to routinizing [56, 58, 59]. Rogers describes two separate DOI processes: one for organizations and another for individuals. This is in contrast to current practice in healthcare organizations, where new interventions or services are often developed solely by matching the proposed innovation to the work needs and characteristics of its intended users [60]. DOI theory, on the other hand, emphasizes the complexity of communication channels and structural aspects of a social system in establishing the conditions that will favor DOI. The details of the ADAPT-IT^3^ process are described in Table 1, with the DOI framework for organizations and individuals described in Table 2. We detail the key steps of the implementation of the proposed randomized controlled trial below.

**Table 1:** Study design and overview of approach to on-site bundled rapid HIV and HCV testing

	ADAPT-IT^3^ Steps	Participants			Data Collection	Method	Output
		Patients	Providers	Managers			
(Intervention Development)	(1) Assessment	30 to 40	10 to 14	3 to 5	Focus group*s*	Focus group discussion with patients and providers separately. Capacity assessment with program managers	Not applicable (n/a)
	(2) Design	n/a	n/a	n/a	n/a	Investigative team reviews assessment data and then devises preliminary approach to developing and refining bundled rapid HIV/HCV testing procedures.	Draft protocol 0 of intervention
	(3) Administration	10	2-4	3-5	“Theater test” (patients observe and respond to demonstration of Draft 0)	Mock intervention is implemented in front of selected patients and providers (from step 1).	Draft protocol 0 of intervention
	(4) Production	n/a	n/a	n/a	n/a	Exit survey follows performance, informs open discussion about modification.	Draft protocol 1 of intervention
	(5) Topical experts	n/a	n/a	n/a	n/a	Investigative team incorporates feedback from theater tests into testing algorithm and manual, develops quality assurance plan.	Draft protocol 1 of intervention
	(6) Integration	n/a	n/a	n/a	n/a	Integrate content from patient/program advisors, and topical experts.	Draft protocol 2 of intervention
(RCT)	(7) Training	n/a	2 to 4	3 to 5	n/a	Train program and study staff to implement draft protocol.	Draft protocol 2 of intervention
	(8a) Testing (Pilot)	20	2 to 4	3 to 5	Exit interviews	Pilot with patients who are representative of target population. Feedback through exit interviews with patients, and feedback from managers and providers who observed the pilot. Produce draft three based on pilot. Refresher training, as appropriate, for program and study staff on draft three of the intervention protocol.	Draft protocol 3 of intervention
	(8b) Testing (RCT)	239	2 to 4	3 to 5	Baseline + follow-up assessments	Test draft three of the protocol Analyze RCT results to determine efficacy.	Assessment of study outcomes
(TRANSLATION)	(9) Translation	20 to 30	5 to 10	3- 5	Focus groups	Elicit reactions of RCT participants. Classify perceived barriers/facilitators to acceptance of bundled rapid testing, referral, and linkage services (patients); and adoption of bundled rapid testing and linkage services (providers and managers).	Multilevel diffusion strategy

**Table 2:** Diffusion of innovation: organization and individual patient level processes

Diffusion of innovation (on-site bundled rapid HIV/HCV testing strategy) process in substance use disorders treatment programs			Innovation-decision process at the individual patient level		
Stages	Description	Sample of questions guiding each stage	Steps	Description	Sample of factors guiding each step
Agenda setting	Identifying organizational challenges that create a need to increase HIV/HCV testing and receipt of test results among patients	What is the primary motivation to adopt a bundled rapid testing strategy?	Knowledge	Patients are introduced to a bundled rapid testing strategy but do not have detailed information about it	Patient knowledge of what bundled rapid testing is, how it works, and why it is beneficial.
Matching	Identifying how the bundled HIV/HCV testing strategy addresses the organizational challenge	What problem or need in the program matches a bundled rapid testing strategy?	Persuasion	To what extent are patients interested in bundled rapid testing, linkage to care, and further information?	Concerns of positive results for one or both HIV/HCV, timing and readiness to test, apathy, and risk perceptions
Redefine/restructure	Modifying a new testing and linkage strategy to fit the organization and reconfigure organizational structures	How would the program operationalize the decision to adopt bundled rapid testing? How would structures be modified to fit the strategy?	Decision	Patients consider advantages and disadvantages and decide to accept bundled rapid testing or not	Discovery of results on engagement in drug treatment. Concerns about confidentiality, access to treatment if HIV- and/or HCV-positive
Clarifying	Stabilizing of the relations among the testing, post-test counseling, and linkage strategies and the organization	What infrastructures would support diffusion? How would the testing strategy be reinvented, if at all?	Implementation	Patients accept bundled rapid testing and determine usefulness of the strategy.	Preferred testing method, concerns about test accuracy, wait time for results, and counseling
Routinizing	Making bundled rapid HIV/HCV testing, post-test counseling, and linkage services a normal part of the organization’s activity.	Could bundled rapid testing become part of a program’s routine? What are the indicators that support the potential for routinizing?	Confirmation	Patients finalize decision to continue to use bundled rapid testing strategy, initiate, and continue care.	Additional information that may influence the decision to use or not use the strategy in the future

### Study facilities and setting

The study will be conducted at Promesa Inc. of the Acacia Network, one of the largest community based SUD treatment programs in the Bronx in New York City [61]. It has the capacity to serve more than 1,700 patients at any point in time across all of its facilities. We will include (1) one facility, which provides methadone to abstinence treatment to patients with opioid dependence (methadone-to-abstinence ambulatory - MTAA). Methadone to abstinence is a medical treatment protocol where methadone is provided in gradually decreasing doses until the point of abstinence. We will also include (2) one facility, which provides behavioral treatments (for example, cognitive behavior approaches) to patients with addictions to opioids and other types of drugs (chemical dependence outpatient program – CDOP. Both MTAA and CDOP are co-located with a federally qualified health center that provides primary healthcare services. The Primary Care Health Center (PCHC) offers on-site laboratory-based enzyme immunoassay (EIA) HIV and HCV testing with delayed results. SUD treatment patients who test positive for either HIV or HCV are referred to relevant medical services. Typically, patients choose to stay in the FQHC co-located with MTAA and CDOP, but if they prefer, they can be referred to another FQHC or primary care health center of their choice.

### Existing evidence-based rapid HIV testing interventions

Our starting point for the development of the on-site bundled rapid HIV/HCV testing intervention is grounded on existing HIV EBIs, which address testing procedures and linkages to care. The rapid HIV testing EBI we will adapt was shown to be highly effective in increasing receipt of HIV test results compared to referral for off-site HIV testing (80 % versus 18 %, respectively) [25]. Patients in this EBI first receive verbal information about the rapid HIV test, including a description of the rapid testing procedure, timing for and meaning of test results, and an explanation of the window period during which an antibody test might be negative despite the presence of HIV infection. Patients are then offered a rapid HIV test [62]. Our approach to linkage to care services will be based on a modified Anti-retroviral Treatment and Access to Services (ARTAS) and Hepatitis C Care Coordination Model (Hep-C CCM) [63–66]. ARTAS is an individual-level, multisession, time-limited intervention with the goal of linking recently diagnosed persons with HIV to medical care soon after receiving a positive test result. ARTAS led to significantly higher linkage to HIV care compared to a “passive referral” standard of care [63, 64]. Hep-C CCM is a case manager-coordinated service that links patients to primary care and hepatology clinics. The intervention was efficacious in increasing both attendance at HCV clinical evaluations and knowledge of HCV [65, 66]. The protocol for on-site bundled rapid HIV/HCV testing will incorporate elements from these interventions. It will be developed through a combination of in-depth interviews, focus group discussions, interactive testing, and expert consultations (see Table 1).

### Study intervention

**Fig. 1:**
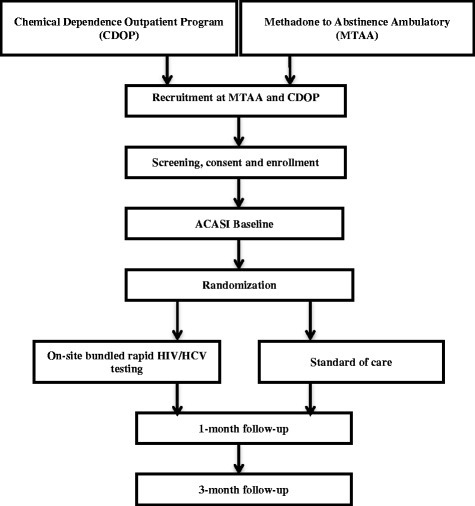
Flow diagram of the trial design: on-site bundled rapid HIV/HCV testing randomized controlled trial (RCT). The diagram illustrates the progression of participants through the different points of the study

Patients randomized to the intervention group will thus receive an offer to participate in on-site bundled rapid HIV/HCV testing. Those who accept this offer will receive pre-test information about testing procedures and education, be tested and receive results during the same visit as testing, and be provided post-test counseling and support services according to the protocol developed through our ADAPT-IT^3^ process. Patients with reactive HIV and/or HCV antibody test results will be actively linked immediately to a healthcare provider for further evaluation and confirmatory testing (see Fig. 1). Patients randomized to the control group will receive the standard of care (SOC). The SOC is venipuncture whole blood laboratory-based HIV and HCV testing with delayed results (enzyme immunoassay). Patients may not receive pre-test information, results, and post-test counseling, especially if non-reactive or negative test result(s). Linkage to care in the SOC is accomplished by referral to a healthcare provider within the participating SUD treatment organization or by passive referral to other health facilities. After the end of the follow-up period (see below), all patients in the control group will be offered access to the on-site bundled rapid HIV/HCV testing intervention. This will include all procedures (that is, rapid HIV/HCV testing, post-test counseling, and linkages to care), as appropriate. Trained study staff will obtain written informed consent from all patients prior to enrollment. Additionally, trained supervisors will monitor the safety and efficacy of the trial intervention on a regular basis. They will ensure that all study procedures are conducted and that data are generated, documented, and reported in compliance with the protocol and applicable regulations.

### Randomization

Participants will be assigned randomly to the intervention or to the control group at the time of the baseline visit. Randomization will be stratified by recruitment site (MTAA versus CDOP programs), location of primary care provider (study site versus other), and type of drug use (injection as a method of drug use versus non-injection drug use). The randomization will be done in blocks of randomized size (varying from four to 10) with computer-generated random numbers. This procedure ensures that the distribution of treatment site and type of drug use will be balanced across intervention and control groups. It also prevents study staff from learning the randomization pattern, which becomes obvious in a fixed block-size scheme. The study statistician will generate the randomization lists, which will be concealed in sequentially numbered, opaque, sealed envelopes with a signature across the sealing point. Trained research assistants, who are blind to the randomization schedule, will provide the random assignment to participants at the time of randomization.

### Eligibility and recruitment

Participants must be (1) able and willing to provide informed consent, (2) seeking or currently receiving SUD treatment services (excluding alcohol only) at the participating treatment programs, (3) at least 18 years old, (4) able to communicate in English, (5) willing to sign a release form that will allow medical record review (to corroborate self-reports of receipt of test results), (6) willing to provide locator information, (7) self report being HIV and HCV negative, or report not knowing their HIV and HCV status, and (8) not have received results of an HIV or HCV test initiated within the last 12 months. We will employ several recruitment strategies to facilitate enrollment to reach our target sample size, including trained research staff, posters, and collaborating with staff of the study site to identify interested patients. We will promote participant retention by collecting locator information, which will be used to contact participants to remind them of follow-up visits and to locate participants who cannot be reached. Participation in the study is voluntary, and participants may choose to end their involvement at any time.

### Study assessments

There will be three assessments during this trial of on-site bundled rapid HIV/HCV testing. These will include a baseline assessment administered prior to randomization, a follow-up assessment conducted 1 month after randomization, and a final assessment conducted 3 months after randomization (see Fig. 1).

### Primary outcome

The primary outcome will be self-reported receipt of HIV and HCV test results. The primary outcome will be assessed one-month post-randomization. Our use of self-reported receipt of test results is consistent with the use of self-reports as the primary outcome for evaluating rapid HIV testing interventions [25].

### Secondary outcome and other study measures

We will also evaluate several secondary outcomes. These will include an additional measure of receipt of test results based on medical records. Study participants will complete release forms (as applicable) in order to grant permission to study staff to review their medical records, including HIV and HCV testing records. Other secondary outcomes will include self-reported sexual risk behaviors and drug use practices such as (i) counts of unprotected vaginal and anal sex acts in the past 3 months with any sex partner, (ii) use of any drugs in the past 3 months, and (iii) unsafe injection practices. Additional measures will evaluate linkage to care by measuring the proportion of patients who initiated HIV care or HCV care before the 3-month follow-up among those whose test results were positive for HIV and/or HCV. Linkage to care for HIV or HCV will be measured separately, to account for variations in barriers to initiating care, which may be greater for HCV than barriers to initiating HIV medical care [67].

### Data sources

In order to measure all outcomes, indicators, and covariates, each study assessment will collect detailed data from study participants. We will use audio computer-assisted self-interview (ACASI) to limit social desirability biases in reporting sensitive behaviors (for example, sexual behaviors) [68]. Interviewers will be trained in the use of the ACASI program and survey to assist respondents as needed. To protect against the risk of loss of confidentiality during and after the trial, all data will be maintained on a password-protected local server. Study datasets will be de-identified prior to analysis.

We will collect the following information:

Demographic characteristics of participants (at baseline), which will include age, sex, race and ethnicity, years of formal education, income, employment status, health insurance, living arrangement including homelessness, number of children (under 18), and history with the criminal justice system (that is, arrests and incarcerations). This information will be used to describe the study sample, to assess for any differences between intervention groups, and to test for selective attrition.

Sexual behaviors: We will collect data on the total number of sex partners in the past 3 months, total number of vaginal sex partners and anal sex partners, total number of unprotected vaginal and total number of anal sex partners, and total acts of unprotected vaginal/anal sex.

Drug use: We will ask about the frequency, duration, and amount of substance use, including alcohol, cannabis, methamphetamine, cocaine, heroin, club-drugs, and mis-used prescription drugs. We will assess injection drug use, including the types of drugs injected and the sharing of needles and other drug paraphernalia (for example, spoons and cotton balls). We will also ask about overdoses and treatment for overdose (that is, the use of naloxone). At baseline, we will measure these behaviors over the 3 months before the interview. These measures will be repeated at 1- and 3-month follow-ups, with adjustments to the recall period.

Utilization of drug treatment services: We will ask the following three questions at baseline and at both follow-up visits: (1) Are you in treatment right now? (2) What treatment are you undergoing? (for example, detox) and (3) How many days have you been in this treatment?

Linkage to medical care: We will also collect data on initiation of treatment among HIV and/or HCV positive patients, using the following questions: (1) Did you have an appointment with a medical provider for HIV services? (2) Did you have an appointment with a medical provider for HCV services? (3) Number of medical appointments made and appointments attended since the last interview, as well as initiation of ART and HCV treatment?

### Power and sample size

We designed the trial to estimate the effects of on-site bundled rapid HIV/HCV testing on receipt of test results separately in each of the two types of SUD treatment facilities (MTAA and CDOP). Based on preliminary data from the study sites for the standard of care, we assumed that 50 % of control group participants enrolled at the CDOP facility will receive their HIV and HCV results after 1 month. We assumed that 55 % of control group participants receive their HIV and HCV test results after 1 month in the MTAA program, which treats a higher proportion of PWIDs. Based on findings from the evidence-based rapid HIV intervention that informs this study [25], we assumed that 80 % of patients in the intervention group would receive their HIV and HCV test results within 1 month of randomization. To detect such intervention effects on the basis of a two-sample, chi-squared test for independent proportions with 80 % power and α = 0.05, we will require 78 patients in CDOP and 108 patients in MTAA facilities. We assumed an 80 % follow-up rate among participants enrolled at the MTAA program. We assumed a slightly lower follow-up rate among participants enrolled at the CDOP facility (75 %) because adherence to behavioral SUD treatment is lower among CDOP patients at Promesa Inc. These are conservative assumptions. For example, during the trial of the rapid HIV testing EBI, which informs this study, researchers achieved a 98 % 1-month follow-up rate [25]. In total, we obtained a total sample size of 239 participants (104 in CDOP and 135 in MTAA).

### Empirical analysis

The primary outcome is a categorical variable denoting self-reported receipt of rapid HIV and rapid HCV test results at 1-month post-randomization. We will use a generalized estimating equation model, which includes covariates for type of drug use (injection/non-injection) and location of primary care provider (study site versus elsewhere). The model will also include indicator variables for HIV/HCV testing intervention status and the type of SUD treatment facility (MTAA versus CDOP), where the participants were recruited. The primary test of our study hypothesis will be a two-degree of freedom test of significance of the intervention across the two types of SUD treatment facilities. In post-hoc tests, the efficacy and effect size in each type of SUD treatment facility will be examined. To do so, we will include an interaction between variables identifying study groups (intervention versus control) and type of SUD treatment facility (MTAA versus CDOP). The primary analysis will be intent-to-treat, where everyone is included in the study group to which they were randomized, regardless of receipt of intervention. In sensitivity analyses, participants who have died or who have been lost to follow-up will be counted as not having tested. We will adjust for baseline covariates that may be associated with attrition from the study sample using inverse probability reweighting procedures as recommended for the GEE model [69].

The secondary outcomes related to linkage to HIV and HCV care, as well as to drug use, are categorical variables. They will be analyzed using techniques similar to those used for the primary outcome. This is also the case for indicators related to HIV and HCV prevalence among patients tested by bundled rapid testing versus standard of care. The number of unprotected sex acts over a period of time is a count variable and will be modeled using Poisson or negative binomial regression (if the variable is over-dispersed) or the zero-inflated version of these if necessary. Type of drug use (injection/non-injection) and the recruitment site (MTAA or CDOP) will be included as covariates. If other covariates predict missing data, they will also be included as control variables. All analyses of secondary outcomes will focus on the pooled data, that is, CDOP + MTAA programs. Because of the limited sample size, we will not conduct formal mediation analyses.

### Translation

We will conduct formative research on the barriers and facilitators of the adoption, implementation, and sustainability of on-site bundled rapid HIV/HCV testing in SUDs treatment programs. To do so, we will collect qualitative data from three groups: patients, providers, and program managers who participated in the randomized trial. This will consist of two focus group discussions (FGDs) with each group, followed by a short questionnaire on individual perceptions. FGDs will be informed by an integrated conceptual framework, derived from the “Diffusion of Innovation” theory [56]. We will identify facilitators and barriers to scaling-up the bundled rapid HIV/HCV strategy, linking infected patients to medical care, and we will develop strategies to address these barriers.

### Ethics

The Columbia University Medical Center (CUMC) Institutional Review Board (IRB) will provide ethical oversight for the study. Ethical approval for this study has been granted from the CUMC IRB (Protocol Number: IRB-AAAN5869).

### Publications

The final results from the study will be submitted for publication in a peer-reviewed journal. We will also disseminate our findings to the study site and study population.

## Discussion

This study is one of the first to systematically develop and test an on-site bundled rapid HCV and rapid HIV testing strategy, complemented with post-test counseling and linkage to care services, to address the overlapping HIV and HCV epidemics among persons with SUDs. It is also novel in employing an efficacy-effectiveness-implementation hybrid design. We hypothesize that the bundled testing strategy will increase HIV and HCV testing rates and knowledge of infection status among persons with SUDs. On-site bundled rapid testing promises significant benefits in terms of both HCV and HIV-related outcomes. We anticipate that the bundled rapid testing strategy will considerably increase HCV testing rates among patients of SUD treatment programs, possibly in proportions similar to those observed during the multi-center trial of rapid HIV testing in such programs [25]. This has the potential to increase HCV case finding and reduce HCV transmission among patients of SUD treatment programs, through the adoption of safer injection and sexual practices. It may also promote treatment initiation, particularly in a context where numbers of patients may gain access to health insurance through the Affordable Care Act (ACA) and new, safe and effective treatments options for HCV have become available [42–45].

Bundled rapid testing may also provide important benefits in terms of HIV prevention and treatment. Since HIV and HCV often transmitted in the same needle-sharing and/or sexual networks, [70–77] testing positive for HCV may serve as a) an early warning system that a person is at a high risk of becoming infected with HIV and b) a system to allow the delivery of intensified risk reduction interventions and to encourage the adoption of protective behaviors (for example, needle exchange, partner reduction, and PrEP). In addition, knowledge of HCV status may improve clinical decisions related to HIV/AIDS care and treatment, including earlier diagnosis of co-infection, improved prevention of liver disease, and better management of side effects. The proposed study will address key barriers that prevent the broad diffusion of bundled rapid testing, including organizational and patient-level barriers, and provide preliminary data for future implementation research on the adoption of evidence-based practices in SUD treatment programs. In particular, it will yield insights about a multicomponent, multilevel intervention designed to foster the adoption and implementation of bundled rapid testing in such settings.

### Trial status

The trial is at the planning stage, with the first six phases of the ADAPT-IT^3^ process already completed. Recruitment of study participants is tentatively scheduled to commence in March 2016.

## Abbreviations

ACA: Affordable Care Act
ADAPT-ITT: Assessment-Decision-Administration-Production-Topical experts-Integration-Training-Testing
ADAPT-IT^3^: Assessment-Design-Administration-Production-Topical experts-Integration-Training-Testing-Translation
ARTAS: Anti-retroviral Treatment and Access to Services
CDC: Centers for Disease Control and Prevention
CDOP: chemical dependence outpatient program
CLIA: clinical laboratory improvement agreement
DOI: diffusion of innovation
EBI: evidence-based interventions
EIA: enzyme immunoassays
FQHC: federally qualified health center
HCV: hepatitis C virus
Hep-C CCM: Hepatitis C Care Coordination Model
HIV: human immunodeficiency virus
MTAA: methadone-to-abstinence ambulatory
PrEP: pre-exposure prophylaxis
PWID: person who injects drugs
RCT: randomized controlled trial
SOC: standard of care
SP: standardized patients
SUD: substance use disorder.
